# A systematic review of grandparents’ influence on grandchildren’s cancer risk factors

**DOI:** 10.1371/journal.pone.0185420

**Published:** 2017-11-14

**Authors:** Stephanie A. Chambers, Neneh Rowa-Dewar, Andrew Radley, Fiona Dobbie

**Affiliations:** 1 MRC/CSO Social and Public Health Sciences Unit, University of Glasgow, Glasgow, United Kingdom; 2 Usher Institute, University of Edinburgh, Edinburgh, United Kingdom; 3 Directorate of Public Health, NHS Tayside, Dundee, United Kingdom; 4 Faculty of Heath Sciences and Sport, University of Stirling, Stirling, United Kingdom; TNO, NETHERLANDS

## Abstract

Many lifestyle patterns are established when children are young. Research has focused on the potential role of parents as a risk factor for non communicable disease in children, but there is limited investigation of the role of other caregivers, such as grandparents. The aim of this review was to identify and synthesise evidence for any influence grandparents’ care practices may have on their grandchildren’s long term cancer risk factors. A systematic review was carried out with searches across four databases (MEDLINE, Embase, Web of Science, PsycINFO) as well as searches of reference lists and citing articles, and Google Scholar. Search terms were based on six areas of risk that family care could potentially influence–weight, diet, physical activity, tobacco, alcohol and sun exposure. All study designs were included, as were studies that provided an indication of the interaction of grandparents with their grandchildren. Studies were excluded if grandparents were primary caregivers and if children had serious health conditions. Study quality was assessed using National Institute for Health and Care Excellence checklists. Grandparent impact was categorised as beneficial, adverse, mixed or as having no impact. Due to study heterogeneity a meta-analysis was not possible. Qualitative studies underwent a thematic synthesis of their results. Results from all included studies indicated that there was a sufficient evidence base for weight, diet, physical activity and tobacco studies to draw conclusions about grandparents’ influence. One study examined alcohol and no studies examined sun exposure. Evidence indicated that, overall, grandparents had an adverse impact on their grandchildren’s cancer risk factors. The theoretical work in the included studies was limited. Theoretically underpinned interventions designed to reduce these risk factors must consider grandparents’ role, as well as parents’, and be evaluated robustly to inform the evidence base further.

## Introduction

Many lifestyle patterns are established when children are young. This is especially true for diet and physical activity patterns [1, 2], two lifestyle areas where there is substantial evidence for the important influence of parental and other role models [3–7]. For a practice such as smoking, it is during the teenage years that this habit is generally established [8]. Smoking, diet and physical activity, along with excess weight, have been identified as risk factors for non communicable disease, particularly cancer [9]. Cancer is the leading cause of death in many countries in Western Europe [10], however, up to 40% of cancer cases could potentially be prevented through healthier lifestyles [9]. Research evidence on cancer prevention is limited compared with that of cancer treatment, however, there is evidence to suggest that exposure to risk factors in childhood increases an individual’s likelihood of cancer morbidity or mortality in adulthood [11–16].

Factors associated with children’s long term cancer risk are first experienced within the family setting. The nuclear family of a father, mother and their children, has been the dominant family model in Western societies in more recent generations [17]. Nevertheless, changes in social conditions, such as an increase in lone parenting, more women in the workforce and prohibitive childcare costs, have led to an increased focus on the role of grandparents’ in their grandchildren’s lives. Health improvements have resulted in greater life expectancy enabling grandparents to support their families by providing childcare, or spending more time with their grandchildren as they are growing up.

Around one fifth of 0–12 year olds in Australia [18], and a quarter of pre-school children in the US [19], are regularly cared for by grandparents. In the UK, it is estimated that grandparent care saves parents around £1700bn per year in childcare costs [20]. The extent of grandparent involvement can vary based on cultural and societal differences. For example, when comparing childcare practices across Europe, Hank and Buber [21] found that grandparents in Greece, Italy and Spain were more likely to provide regular childcare to their grandchildren, and that grandparents in the Netherlands, France and Nordic countries were least likely to. These patterns reflect the differing social and cultural contexts in these countries, such as labour market participation by older women and state provision of formal childcare [22]. Within the UK, the important childcare role that grandparents provide has been recognised at government level with grandparents caring for grandchildren entitled to receive National Insurance Credits towards their state-provided pension [23]. Forthcoming legislation will also allow grandparents to share parental leave with parents in a child’s first year of life, and for employed grandparents to have the right to work flexibly to allow them to care for their grandchildren [24].

There is a significant literature around the impact of caring for grandchildren on grandparents’ health, particularly when grandparents are called upon to become primary carers to grandchildren. While some studies indicate that caring for grandchildren can have an adverse impact on grandparents’ health [25–27], there is evidence that after controlling for sociodemographic factors, this caring role can have a beneficial impact on physical health [28]. It is less clear how grandparents’ care influence their grandchildren’s health. While there is some evidence that grandparents can play a significant role in supporting their grandchildren’s social and emotional wellbeing [29, 30], there is also evidence that the multifaceted nature of these relationships can have both beneficial and adverse impacts [31, 32].

The influence of grandparents’ care practices on grandchildren’s physical health is also unclear, particularly for non-communicable diseases like cancer which are more likely to be experienced later in their grandchildren’s lives. With greater recognition of the key role of grandparents in grandchildren’s lives, there have been calls for parenting advice to be broadened to encompass grandparents. For example, in Scotland, this has been proposed as a potential strategy to help tackle health and educational inequalities in communities with high levels of disadvantage [33]. There is therefore a need to identify, review and synthesise the literature on grandparents’ influence on their grandchildren to inform practitioners, policy makers and academics further about family dynamics that impact on health outcomes.

The aim of this systematic review was to identify and synthesise evidence for the influence of grandparents on their grandchildren’s long term cancer risk factors. Key objectives were:

1. To examine the availability of evidence for grandparents’ influence on a range of grandchildren’s cancer risk factors;
2. To identify whether this evidence indicates that grandparents have a beneficial or adverse impact on grandchildren’s cancer risk factors.
3. To identify theoretical frameworks used to inform research in this area.

## Methods

The breadth of factors considered in the review was determined via Cancer Research UK’s research on key preventable risk factors for cancer [9]. Cancer risk factors applicable to children that could potentially be influenced by grandparents were selected. These included tobacco (smoking or exposure), obesity, diet, alcohol consumption, sun exposure and inactivity. PRISMA guidelines were followed in reporting this review [34].

### Search strategy

Searches were carried out using defined terms in Web of Science, Medline, Embase and PsycInfo from database start dates to May 2017. The research team hand searched the reference lists of included papers, and citing papers, and carried out a search of Google Scholar using variations of the search terms in S1 Table.

### Search terms

Synonyms were identified around the Population (grandparents and grandchildren) and Outcome (cancer risk factors) components of the PICOS framework (see S1 Table). MeSH terms and subject headings were used where appropriate. The Boolean operator OR was used to combine within Population and Outcome search results, with AND used to combine these two blocks, to search titles and abstracts.

### Selection of articles

All study years and designs were included if the relevant risk factors were examined. A further inclusion criterion was that publications must provide an indication that grandparents spent time with their grandchildren, either by providing childcare, living with children or during visits. Exclusion criteria included grandparents who acted as primary caregivers, grandchildren with serious medical conditions, and studies examining grandparents’ health outcomes only. Abstracts, newspaper reports and non-English language publications were also excluded.

### Quality assessment

Study quality was assessed using National Institute for Health and Care Excellence checklists relevant to the particular study design [35]. For quantitative studies (including observational, cohort and intervention studies), quality assessment focused around the representativeness of the study population, the method of selection, reliability of outcomes, and appropriate analyses. For qualitative studies, assessment was made based on aim, design, data collection, researcher role, description of context, appropriate methods and analysis, richness of findings and conclusions. Assessment of review articles was based on a focused question, relevance of included studies, rigour of search, study assessment and appropriately described methods. An overall study quality measure was also provided (high, medium or low) based on scores for the individual components assessed in each of the studies.

### Data extraction

Data was extracted using a predefined form adapted from the Cochrane Collaboration [36]. Extracted data included study geography, participant characteristics, sample size, study aim, theoretical framework, outcome measures, main findings, and for qualitative data, participant quotations and author syntheses that discussed grandparent impact on grandchildren for the relevant risk factors.

### Data synthesis

Given the range of potential outcome measures in the included quantitative studies, meta-analysis of data was not carried out. Instead, grandparent impact was defined as beneficial, adverse, mixed (some beneficial and some adverse outcomes) or as having no impact for all study types. Qualitative data then underwent further synthesis through a thematic analysis of both participants’ quotations and article syntheses. Extracted text was read and reread by two reviewers. They each identified key themes and shared these with all study authors. Final themes were then agreed upon, and text coded under each theme. Themes were reorganised and structured hierarchically where possible.

## Results

**Fig 1:**
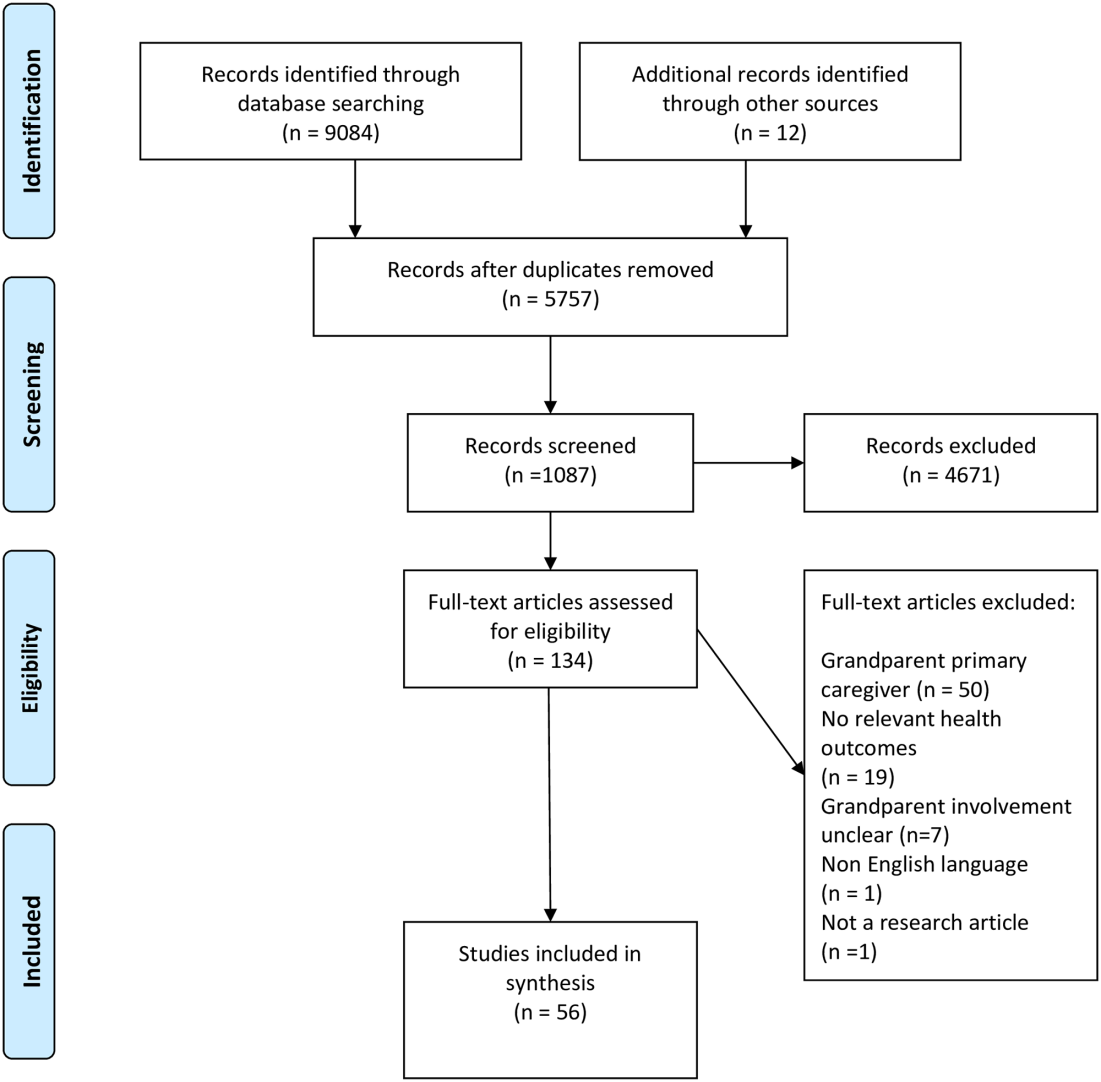
Flow diagram of search results.

The searches yielded 5745 publications after removal of duplicates (Fig 1). All titles and abstracts were screened by two reviewers, with a third reviewer providing advice when disagreements on inclusion arose. This resulted in 134 publications retrieved for full text inspection, and 44 included in the analysis. A further 12 were retrieved from reference list and Google Scholar searches. A total of 56 publications were included. Explanations for exclusion of studies at full text stage were no indication of grandparents spending time with their grandchildren, or grandparents being primary caregivers, and no focus on children’s cancer risk factors.

No studies examined sun protection. A single study examined alcohol [37]. There was evidence examining grandparent impact on tobacco smoking or exposure [37–52] (n = 16), weight [32, 53–68] (n = 17), diet [57, 61, 65, 66, 69–89] (n = 26), and physical activity [52, 57, 58, 61, 65, 71, 76, 79, 90] (n = 9). Study details for publications examining weight, diet and physical activity are discussed together, although the main study results are discussed separately for each of these areas. Tobacco studies are discussed separately, as is the single alcohol study. Study details can be found in Tables 1–4.

**Table 1:** Overview of weight studies.

Study (Name, Year, Country, Quality rating)	Study aims	Sample	Study design	Outcome of interest/ theoretical framework	Main findings
Sowan & Stember (2000) [62]	To facilitate an understanding of the influence of the parental characteristics on the development of infant obesity.	630 infants (15 months) and their families. Recruited from six health care study sites.	Longitudinal prospective design: 1 months; 4 months; 7 months; 10 months; 14 months	BMI Obesity	Impact: no impact Grandmother living in the home was not significant predictor of child obesity.
USA					
High quality				Web of causation model	
Jain et al. (2001) [56]	To explore mothers’ perceptions about how they determine when a child is overweight and what barriers exist to prevent or manage childhood obesity.	18 low income mothers of preschool children (13 black and 5 white) who were at risk for later obesity.	3 focus groups	Weight	Impact: adverse Grandparents (on the whole) more permissive–causes difficulties–mothers feel undermined.
USA					
High quality			Thematic analysis (though didn’t explicitly state this)		
Gao et al. (2007) [53]	To identify effective obesity interventions in the Chinese literature.	3 Chinese and 9 international databases. Studies with: Intervention ˃3 months Control group and anthropometric measures	Systematic review	Interventions to reduce overweight & obesity. Studies that evaluated public health programmes aiming to prevent, control or reduce obesity or obesity-related factors in China.	Impact: adverse In Chinese families, many grandparents provide childcare assistance–preference for overfeeding. Grandparents as barrier to interventions.
China					
Medium quality					
Hawkins et al. (2008) [55]	To investigate factors related to early childhood overweight only among mothers in employment.	13,113 parents and children (aged 3 years)	Millennium Cohort Study—Longitudinal	Child overweight (including obesity)	Impact: no impact No difference in early childhood overweight between children cared for by informal arrangements (75% grandparents), and those cared for by their mother /mother’s partner AOR 1.02 (95%CI 0.92–1.13).
UK					
High quality					
Pearce et al. (2010) [59]	To explore the association between childcare and overweight.	Children born in UK between Sept 2000 and Jan 2002. Sweep 1 = 9 months–n = 18296 Sweep 2 = 3 years–n = 14630	Millennium Cohort Study—Longitudinal	Obesity	Impact: adverse Children cared for in informal childcare (75% grandparents) between age of 9 months and 3 years more likely to be overweight than those cared for only by a parent ARR 1.15 (95%CI 1.04–1.27), particularly if in full time care ARR 1.34 (95%CI 1.15–1.57). Increased risk only observed for those in informal childcare full time. Increased risk of overweight only significant in those care for by grandparents.
UK					
High quality					
Pocock et al. (2010) [60]	To synthesise qualitative research concerning parental perceptions regarding behaviours for preventing overweight and obesity in young children.	Qualitative papers with children under 12 as the focus.	Systematic review	Overweight and obesity	Impact: adverse Grandparents undermining parents by allowing children to eat what they want. Wish to avoid conflict with grandparents.
Various countries					
Medium quality					
Glassman et al. (2011) [54]	Latino parents’ perceptions of their ability to prevent obesity in children.	26 Latino parents of preschoolers at an NYC Headstart programme.	3 focus groups	Obesity prevention	Impact: adverse Grandparents providing less healthy food–children know they will get it from grandparents.
USA					
Medium quality			Social cognitive theory used as themes for thematic analysis.	Social cognitive theory	
Watanabe et al. (2011) [65]	To examine the effects of maternal employment and the presence of grandparents on lifestyles and overweight and obesity in Japanese pre-school children	2114 children aged 3–6 years who attended child care facilities and primary care givers.	Cross-sectional survey	Overweight/obesity	Impact: adverse Living in a three generational family associated positively with children’s overweight/ obesity, even after adjustment for maternal employment AOR 1.59 (95%CI 1.08–2.35)
Japan					
High quality					
Pulgarón et al. (2013) [61]	To evaluate the rate of Hispanic children who have grandparents involved in caretaking and whether grandparents’ involvement has a negative impact on feeding practices, children’s physical activity and BMI.	199 Hispanic children and parents from a Miami elementary school (5–12 years).	Cross-sectional survey	zBMI score	Impact: mixed/no impact No difference in zBMI for those who did and did not have a role in grandparent caring. Degree of grandparent involvement not correlated with child’s zBMI. zBMI positively correlated with parent and grandparent disagreement. For other Hispanic (non Cuban) children, grandparent caretaking had lower zBMI.
USA					
Low quality					
Tanskanen (2013) [63]	The association between maternal and paternal grandmothers’ childcare provision and early years overweight in the UK.	3 year old children from 15,109 families but 9000 in sample–where biological mother where living with child and biological father.	Millennium Cohort Study–Longitudinal information but second wave.	Overweight (including obesity)	Impact: adverse Where maternal grandmother provides most childcare, 20% more likely for child to be overweight. Not significant for paternal grandmother but underpowered. No differences based on mothers’ socioeconomic status.
UK					
High quality					
Toftemo et al. (2013) [64]	To explore parents’ views & experiences when health professionals identify their pre-school child as overweight.	Parents of 10 overweight children aged 2.5–5.5 years recruited at well child clinics in rural parts of Norway.	Indepth interviews	Overweight	Impact: mixed Grandparents undermine parents’ efforts to make changes—but some are excellent. Parents wanted support from grandparents. Children can be spoiled eg sweet foods. Need to educate grandparents.
Norway			Thematic analysis (systematic text condensation)		
Medium quality					
Li, Adab & Cheng (2014) [57]	To identify family & neighbourhood environmental correlates of overweight and related behaviour.	Parents of 497 Chinese 8–10 year olds in two Southern cities. Mix of socio-economic school backgrounds.	Cross-sectional study design	Overweight (including obesity)	Impact: adverse Children cared for by grandparents over twice as likely to be overweight/ obese AOR 2.03 (95%CI 1.19–3.47). Children living with at least two grandparents in house higher risk than those living with none AOR 1.72 (95%CI 1–2.94).
China			Routinely collected height and weight data.		
Medium quality					
Li et al. (2015) [58]	To investigate the impact of grandparents on the childhood obesity epidemic in China, in order to inform the development of culturally relevant childhood obesity intervention programmes.	Qualitative study: 25 parents & 24 grandparents of primary school children; 15 teachers & school nurses; 15 school catering staff; 4 head teachers	Mixed methods Focus groups and interviews Cross-sectional survey and measures	Obesity	Impact: adverse/ no impact Grandparents prefer grandchildren to be overweight, have poor knowledge of obesity health consequences and healthy diets, overfeed grandchildren, and limit activity.
China					
Qualitative: High quality					
			Thematic analysis		
Sata et al. (2015) [66]	To examine the effect of caregiver differences on subsequent childhood habituation (between-meal eating habits, being overweight, and BMI).	Parents of children 3 years old in 1992. Follow ups when children aged 6, 12 and 22 (child completed at age 22).	Cohort study	Overweight BMI	Impact: no impact/ adverse Both boys and girls cared for by grandparents more likely to be overweight at age 3, but boys also more likely to be overweight at ages 6 and 12. Grandparent care was also associated with increases in BMI at ages 3, 6 and 12 for boys and girls.
Japan					
Medium quality					
Zong et al. (2015) [67]	To describe a wider spectrum of risk factors for obesity among preschool children (including being cared for by grandparents).	1996–1234 boys; 610 girls 2006–2290 boys; 1008 girls 3–7 year old children attending kindergarten (parents completed questionnaires).	Case control surveys in 1996 and 2006 –children who were obese matched to similar child who was not obese.	Obesity	Impact: no impact/ adverse 1996 –no impact of grandparent care. 2006 –grandparent care increases likelihood of children being obese AOR 1.44 (95%CI 1.05–1.97);
China					
Medium quality					
Ikeda et al. (2017) [68]	To track the likelihoods of childhood overweight and obesity from living in a household with grandparents from early childhood to school age.	43,046 children aged 2.5, followed up multiple times until age 13. Parent-complete until age 11	Cohort study	Overweight & obesity	Impact: adverse/ no impact Living with grandparents increased the odds of boys being overweight or obese from ages 5 to 13, and in girls from ages 5 and 8–12
Japan					
Medium quality					
Pulgarón et al. (2016) [32]	To gather and synthesise research findings on the effects of grandparent involvement on children’s physical health outcomes.	26 papers published between 1994–2014 reporting data on child health, well-being and safety outcomes.	Literature review	Weight	Impact: mixed 5/6 studies found adverse impact of grandparent involvement on child weight.
Various					
Medium quality					

**Table 2:** Overview of diet studies.

Study (Name, Year, Country, Quality rating)	Study aims	Sample	Study design	Outcome of interest/ theoretical framework	Main findings
Auld & Morris (1994) [85]	To identify the range of infant/toddler feeding practices among Anglo & Mexican American adolescent mothers and their mothers.	20 Anglo & Mexican-American adolescent mothers and 20 grandmothers of children 6–24 months.	Indepth interviews	Feeding practices	Impact: adverse Mothers expressed frustration that grandparents provided children with unhealthy foods.
USA					
Medium quality					
Kagamimori et al. (1999) [76]	To assess whether obese 3-year-old children have a greater likelihood of obesity-related lifestyles according to social variables (including living in an expanded family).	8834 parents of 3 year old children born in 1989 in Toyama prefecture Japan.	Cohort study	Irregular snack intake	Impact: adverse Children living in expanded families (ie living with grandparents) were more likely to eat irregular snacks AOR 1.44 (95%CI 1.30–1.59).
Japan					
High quality					
Dixey et al. (2001) [69]	To gain insight into children’s understanding of healthy eating, and to explore the barriers & facilitating factors for dietary behaviour change in children.	300 children aged 9–11 years from 10 schools in Leeds. -145 girls; 155 boys Mixed SES schools	60 Focus groups	Diet–healthy eating	Impact: adverse Children reported grandparents indulged them, creating conflict with parents.
UK					
Low quality					
Green et al. (2003) [72]	To examine socio-cultural familial and environmental factors influencing health, eating habits and physical activity contributing to overweight and obesity.	8 families: 47 children aged 5–15 years; 29 parents; 42 grandparents from Turkish, Greek, Indian and Chinese communities migrating to Australia in last 3 generations.	Semi-structured interviews with key informants 3 generation families and generation-by-generation focus groups.	Eating habits	Impact: adverse Grandparents feel under pressure to provide high sugar and fat foods due to advertising. Wished for educational support–community based.
Australia					
Medium quality					
Jiang et al. (2007) [74]	To investigate how grandparents influence their young grandchildren’s eating behaviours in Chinese 3-generation families	12 parents (3 male) 11 grandparents (4 male) in Beijing selected from 4 kindergartens in 2 different districts. Participants chosen for different income levels, occupational status and place of residence.	Semi-structured indepth interviews	Young children’s eating behaviours	Impact: mixed Grandparents important role in preparing family meals–cook most of them. Grandparents’ attitudes influence young children’s nutrition & eating habits. Overfeeding and urging to overeat–feel it shows they are doing a good job. Grandparents use food as an educational and emotional tool. Grandparents’ experiences of poverty. Conflict between generations.
China					
			Thematic analysis		
Medium quality					
Kaplan et al. (2006) [77]	How youth, parents, and grandparents discuss eating healthy/ unhealthy and identify intergenerational strategies for educators to improve this presentation.	44 in total: 21 pre-teens; 16 parents; 7 grandparents from Pennsylvania. Nutrition education program sites (serve low income & multi-generational populations). All grandparents prepared meals and snacks for grandchildren at least 3 times per week.	3 focus groups with 4–8 families.	Eating healthfully and unhealthfully.	Impact: beneficial Grandparents attributed asthma in grandchildren to weight brought on from eating junk foods and over eating. Grandparents proactively managed food entering house, proactively managed foods, involved children in food-related activities. Grandparents spoke with children about healthy eating. Grandparents tried to accommodate children. Felt they did not have ability to limit children’s unhealthy eating habits.
USA					
Low quality			Thematic analysis		
Styles et al. (2007) [82]	To identify obesity related concerns of Hispanics, Black & White parents of young children (5–8 years)	54 black, white & Hispanic parents with children 5–8 years. 37 mothers; 17 grandmothers. Needed to have at least one child with ‘weight problem’ identified by parent or physician. 56% high school education or less. 46% working full-time. 47% annual incomes below $20k.	8 Focus groups: 2 Hispanic; 3 black; 3 white.	Diet	Impact: mixed Grandparents love to see children eat–concern about snacks. Grandparents giving in to children when providing childcare. Instance of one grandmother trying to work with the other to provide healthier food.
USA					
Medium quality			Content and thematic analysis	Examined intervention suggestions using socio-ecological approach.	
Dwyer et al. (2008) [70]	To explore parents’ experiences & challenges in supporting healthy eating & physical activity among their pre-school children.	39 parents from 3 childcare centres in Ontario with a child aged 2–5 years. 34 Female, 5 male; 32 Caucasian, 2 Chinese; 24 completed University or college.	5 focus groups	Healthy eating	Impact: adverse High fat/sugar foods when grandparents care for children.
Canada					
High quality			Constant comparative method.	Used socio-ecological model to discuss.	
Lindsay et al. (2009) [79]	To describe immigrant Latina mothers’ perceptions of factors that act as barriers for establishing healthy eating and PA habits of their pre-school children.	Low income Latina mothers in North East US (n = 31) with a baby 48 months or less. Every 5^th^ woman involved in an RCT.	6 focus groups and 20 indepth interviews.	Eating healthily	Impact: adverse Example of grandmother wanting to see granddaughter chubby.
USA					
High quality			Content analysis		
Speirs et al. (2009) [81]	Are grandmothers involved in purchasing food for or feeding preschool grandchildren? What resources do both have to purchase fruit and vegetables do they allow them to buy a healthy amount? Do mothers and grandmothers consume fruit and vegetables and understand their importance?	62 low income mothers (n = 44) and grandmothers (n = 18) rural Maryland with pre-school children/ grandchildren.	Cross-sectional survey	Fruit and vegetable consumption	Impact: mixed Grandmothers bought fruit and vegetables however they eat less than recommended.
USA					
Low quality					
Johnson et al. (2010) [75]	To explore the personal relationship between a mother and her mother, grandmother or other female relation and its influence on the present family’s food choices.	7 mothers recruited from the 2008 Brazos Valley Household Food Inventory Study. Low income with at least one child under 18.	First indepth qualitative interview.	Food choices	Impact: mixed Grandparents can have beneficial or adverse impact. Where impact adverse, creates family tensions.
USA			Photo-elicitation and second interview		
High quality					
			Grounded theory and Sift and Sort: Think and Shift		
Roberts & Pettigrew (2010) [80]	To examine influence of family & peer groups on diet. Focused on the social and psychological factors contributing to childhood obesity	163 study participants– 124 children (6–12 years) high SES n = 33 medium SES n = 48 low SES n = 43. 39 parents (primary carers)–fathers (n = 3) High SES n = 13; Medium SES n = 12; Low SES n = 14	Individual and small group interviews Children– 26 individual interviews, 39 small group interviews. Parents– 27 individual interviews, 4 small group.	Diets	Impact: adverse ‘Killing them with kindness’ ‘The Rebel grandparent’ builds grandparent/child relationship Undermining parents Single mothers more reliant
Australia					
Low quality					
Watanabe et al. (2011) [65]	To examine the effects of maternal employment and the presence of grandparents on lifestyles and overweight and obesity in Japanese pre-school children.	2114 children aged 3–6 years who attended child care facilities and primary care givers	Cross-sectional survey	Eating/skipping breakfast Regular meals Fixed snacking	Impact: adverse/ no impact 3 generational families less likely to eat irregular meals—no other areas significant.
Japan					
High quality					
Goh et al. (2013) [88]	To illustrate the bi-directional and dialectical interactions among caregivers and between single children and their multiple caregivers in Xiamen.	33 parents and grandparents 10 3-generational families and parents (n = 20) from 10 nuclear families with single children between 6–9 years.	Indepth interviews with parents and grandparents	Meal time dynamics	Impact: adverse Grandparents feel it is important for children to finish their meals. Higher conflict and tension in 3-generation families. Children in multi-generational families fed by grandparents.
China			Cross-sectional survey		
Medium quality					
			Intergenerational Parenting Coalition (IGPC) as framework for analysis.	Intergenerational Parenting Coalition	
Pulgarón et al. (2013) [61]	Evaluate the rate of Hispanic children who have grandparents involved in caretaking and whether grandparents’ involvement has a negative impact on feeding practices, children’s physical activity and BMI.	199 Hispanic children and parents from a Miami elementary school (5–12 years)	Cross-sectional survey	Negative eating	Impact: mixed/no impact Greater grandparent involvement associated with higher negative eating and more negative eating.
USA					
Low quality					
Wasser et al. (2013) [83]	Is non maternal caregiver involvement associated with breastfeeding, timing of introduction of complementary foods, and dietary intakes among infants and toddlers?	217 low-income, African–American mother–infant dyads, followed from 3 to 18 months postpartum.	Cohort from Infant Care, Feeding and Risk of Obesity Study.	Any intake of juice, fried potatoes, desserts and sweets, sweetened beverages, salty snacks & early complementary feeding. Daily servings of fruit, vegetables, fruit juice, fried potatoes, desserts and sweets, salty snacks.	Impact: no impact/ adverse Only a significant association between grandmother as non maternal caregiver who provides food and child intake of juice AOR 1.97 (95%CI 1.02–3.81).
USA					
High quality					
Farrow (2014) [87]	Whether there are differences between parents’ and grandparents’ feeding practices and whether this is related to number of hours grandparents care for grandchildren.	50 parents 50 grandparents of children 2–8 years. 49 mothers 1 father 39 grandmothers 11 grandfathers	Cross-sectional survey	Comprehensive feeding practices	Impact: mixed Grandparents report using more maladaptive feeding practices eg using food to regulate emotions and restricting food but also providing a healthy food environment. More hours grandparents spent with child, the more practices reflected parents’.
UK					
Medium quality					
Hoare et al. (2014) [73]	To examine factors that influence mothers when choosing drinks for their children	32 mothers of young children from Victoria’s Barwon South Western Region (range of demographics). Children 6–12 months	Semi-structured interviews	Drinks	Impact: adverse Grandparents increased consumption of sweet drinks. Difficult for parents to confront grandparents
Australia					
High quality			Thematic analysis		
Li, Adab & Cheng (2014) [57]	To identify family & neighbourhood environmental correlates of overweight and related behaviour.	Parents of 497 Chinese 8–10 year olds in two Southern cities. Mix of socio-economic school backgrounds.	Cross-sectional study design Routinely collected height and weight data	Weekly consumption of unhealthy snacks and F&V	Impact: adverse/ no impact Children cared for by grandparents have higher consumption of unhealthy snacks ß = 2.13 (95%CI 0.87–3.4). There was no relationship for F&V consumption.
China					
Medium quality					
Boni (2015) [84]	To show how children’s food culture has changed and how it is negotiated in a post-socialist Poland.	15 families (mothers, fathers, grandparents and children). 3 primary schools (principals, teachers, school shop owners, cooks and children)	Ethnographic study	Children’s food culture	Impact: mixed Grandparents participate in rule breaking over unhealthy foods with children. Some grandparents follow parents’ rules.
Poland					
Medium quality				Practice theory	
Kavle et al. (2015) [78]	To gain an understanding of the cultural and contextual influences on nutrition practices in children 0–23 months of age.	150 mothers with children 0–23 months. 40 fathers 40 grandmothers 40 health providers	Indepth interviews:	Diet	Impact: adverse Grandparents provided children with non recommended foods, such as cakes/ biscuits.
Egypt			3 longitudinal.		
Medium quality			interviews with mothers & single interviews with others	WHO framework on Childhood Stunting	

			Thematic analysis		
Mena & Gorman (2015) [89]	To explore 1) precursors and contextual factors that influence parental feeding; 2) parental perceptions and knowledge of the child-care food environment.	36 Hispanic caregivers (34 mothers, 2 grandmothers) of a child 2–5 years enrolled at a child care centre.	4 Focus groups	Parental feeding practices	Impact: adverse Grandparents indulged children with non healthy foods and did not support parents to establish healthy eating habits.
USA					
Medium quality			Thematic and structural coding		
Sata et al. (2015) [66]	To examine the effect of caregiver differences on subsequent childhood habituation (between-meal eating habits, being overweight, and BMI).	Parents of children 3 years old in 1992. Follow ups when children aged 6 and 12.	Cohort study	Between-meal eating	Impact: no impact/ adverse Compared to care by mothers, there was some evidence that daytime grandparents’ care resulted in higher prevalence of between-meal eating before dinner for boys and girls at 6 and girls at 12 years.
Japan					
Medium quality					
Eli et al. (2015) [71]	To elucidate parental and grandparental perspectives on young children’s feeding and physical activity and identify how families negotiate potential differences.	22 mothers and 27 maternal grandmothers of children aged 3–5.	Indepth interviews	Feeding	Impact: mixed Parents perceived grandparents to provide high sugar products excessively. Grandparents believe they provide balance through cooking meals.
USA					
High quality			Thematic analysis	Familial homeostasis	
Eli et al. (2017) [86]	To examine mothers’ and maternal grandmothers’ attitudes, knowledge, and practices regarding preschool aged children’s beverage consumption. To identify intergenerational differences, and consider their potential impact on young children’s beverage consumption habits.	11 mothers and 11 maternal grandmothers of children aged 3–5.	Indepth interviews	Beverage consumption	Impact: mixed Some grandparents ‘spoiled’ children with high sugar drinks, whilst others limited these drinks.
USA			Thematic analysis		
High quality					
Pulgarón et al. (2016) [32]	To gather and synthesise research findings on the effects of grandparent involvement on children’s physical health outcomes.	26 papers published between 1994–2014 reporting data on child health, well-being and safety outcomes.	Literature review	Feeding	Impact: mixed There were mixed results for impact on feeding.
Various					
Medium quality					

**Table 3:** Overview of activity studies.

Study	Study aims	Sample	Study design	Outcome of interest/ theoretical framework	Main findings
(Name, Year, Country, Quality rating)					
Kagamimori et al. (1999) [76]	To assess whether obese 3-year-old children have a greater likelihood of obesity-related lifestyles according to social variables (including living in an expanded family).	8834 parents of 3 year old children born in 1989 in Toyama prefecture Japan.	Cohort study	Physical inactivity	Impact: adverse Children living in expanded families (ie living with grandparents) were more likely to be physically inactive AOR 1.16 (95%CI 1.0–1.27).
Japan					
High quality					
Lindsay et al. (2009) [79]	To describe immigrant Latina mothers’ perceptions of factors that act as barriers for establishing healthy eating and physical activity habits of their pre-school children.	Low income Latina mothers in North East US (n = 31)–baby 48 months or less. Every 5^th^ woman involved in an RCT.	6 focus groups and 20 indepth interviews among immigrant low-income Latina mothers in North East US.	Physical activity	Impact: adverse Day care greater opportunity for exercise than care by relatives.
USA					
High quality					
			Content analysis		
Watanabe et al. (2011) [65]	To examine the effects of maternal employment and the presence of grandparents on lifestyles and overweight and obesity in Japanese pre-school children.	2114 children aged 3–6 years who attended child care facilities and primary care givers.	Cross-sectional survey	Physical activity: Time watching TV Time playing outside	Impact: no impact No significant results.
Japan					
High quality					
Li, Adab & Cheng (2013) [57]	To identify family & neighbourhood environmental correlates of overweight and related behaviour.	Parents of 497 Chinese 8–10 year olds in two Southern cities. Mix of socio-economic school backgrounds.	Cross-sectional study design Routinely collected height and weight data.	Whether child engages in recommended levels of moderate or vigorous activity.	Impact: mixed Children who lived with one grandparent more likely to achieve 60 min of MUPA per day compared with children living with none AOR 2.15 (95%CI 1.05–4.39). No relationship for two or more grandparents AOR 1.26 (95%CI 0.64–2.50).
China					
Medium quality					
Pulgarón et al. (2013) [61]	Evaluate the rate of Hispanic children who have grandparents involved in caretaking and whether grandparents’ involvement has a negative impact on feeding practices, children’s physical activity and BMI.	199 Hispanic children and parents from a Miami elementary school (5–12 years).	Cross-sectional survey	Sedentary activity	Impact: no impact No association between greater grandparent involvement and sedentary activity. Greater disagreement between grandparents and parents associated with increased likelihood of sedentary activity (r = .27, p = .02).
USA					
Low quality					
Lako (2014) [90]	To reveal characteristics and tendencies which appear in the lifestyles of families. How different generations affect each other eg, how they influence each others’ view in connection with a healthy lifestyle & exercise.	Survey: 509 10–14 year olds (294 girls, 210 boys) 509 parents 509 grandparents (371 grandmothers, 115 grandfathers)	Cross-sectional survey and indepth interviews with 150 participants	Physical activity	Impact: beneficial Grandparents supportive of grandchildren’s participation in sport. Grandparents take children to sporting activities or cheer them on.
Hungary					
Low quality					
		Indepth interviews: 50 children 50 parents 50 grandchildren			
Li et al. (2015) [58]	To investigate the impact of grandparents on the childhood obesity epidemic in China, in order to inform the development of culturally relevant childhood obesity intervention programmes.	25 parents & 24 grandparents of primary school children 15 teachers & school nurses 15 school catering staff 4 Head teachers	Mixed methods Focus groups and interviews	Physical activity	Impact: adverse Grandparents limit children’s activity by doing their household chores.
China					
Qualitative data: Medium quality					
Eli et al. (2015) [71]	To elucidate parental and grandparental perspectives on young children’s feeding and physical activity and identify how families negotiate potential differences.	22 mothers and 27 maternal grandmothers of children aged 3–5.	Indepth interviews	Sedentary behaviour Physical activity Screentime	Impact: mixed Inconsistent evidence that grandparents encourage sedentary behaviour. Grandparents’ more lax about screentime rules. Physical activity was an area that raised few differences or tensions.
USA					
High quality			Thematic analysis	Familial homeostasis	
Wang & Qi (2016) [52]	To determine association between family structure and Physical activity of Chinese children aged 10–16.	612 10–16 year olds	Cross-sectional survey and physical activity measures	Physical activity	Impact: adverse Adolescents living with grandparents less active than those not living with grandparents β = -0.17 (P<0.001).
China					
Medium quality					

**Table 4:** Overview of tobacco studies.

Study	Study aims	Sample	Study design	Outcome of interest/ theoretical framework	Main findings
(Name, Year, Country, Quality rating)					
Lam et al. (1999) [42]	Whether passive smoking is associated with respiratory ill health in primary school children.	3964 8–13 year olds 94% response rate 2156 boys 1779 girls	Cross-sectional survey	Respiratory symptoms	Impact: adverse/ no impact Positive association between grandparents’ smoking and 6/17 child respiratory symptoms.
Hong Kong					
High quality					
Hopper and Craig (2000) [38]	To identify sources of Environmental Tobacco Smoke exposure for children attending a hospital based paediatric resident practice.	174 caregivers of children visiting a children’s hospital-based resident practice.	Cross sectional survey Face-to-face	ETS	Impact: adverse Most of children’s exposure to tobacco outside the home occurs in a grandparent’s house.
USA					
Low quality					
Yousey (2007) [39]	To explore families’ attitudes about smoking & their perceptions of the effects of ETS exposure on their children.	20 parents from low income families whose children received healthcare services from school-based health centres– 18 mothers, 2 joint mother/father interviews.	Face-to-face interviews with a semi-structured guide.	ETS	Impact: adverse Parents limiting grandparent contact with children as they smoke around them. Difficult to ask grandparents not to smoke around children.
USA					
High quality			Immersion, coding & detailed description–content analysis.		
Hruba and Zaloudikova (2008) [43]	To document the effectiveness of a no smoking programme with respect to children’s family smoking history.	1423 children from programme and control groups	Cross-sectional survey	Smoking	Impact: adverse/ no impact In families where both parent and grandparent smoke, smoking of women and men criticised by significantly less children (90.8%, 84.6%, p˂0.001). Children whose parents and grandparents do not smoke reported hardly ever meeting smokers. If children had non-smoking parents but smoking grandparents, they did not differ in decision about future smoking from families of non smokers.
Czech Republic					
Low quality					

Carlsson et al. (2010) [44]	To investigate & analyse attitudes to tobacco prevention among child healthcare nurses.	196 nurses working at 92 child healthcare centres in two countries in South-Eastern Sweden (160 returned questionnaires)	Cross-sectional survey Postal	ETS	Impact: adverse One nurse wrote a comment that there are problems with smoking grandparents.
Sweden					
Medium quality					
King et al. (2009) [40]	To examine households with children’s association with adult smoking behaviour to design effective interventions to reduce Second Hand Smoke exposure	46,982 US children 0–18 years	Data from Medical Expenditure Panel Survey 2000–2004 Cross sectional	Child residence in a home with a smoker.	Impact: adverse/ no impact 53% of children who lived in grandparents’ home live with a smoker, 33% with parents. AOR 1.22 of living with a grandparent compared with living in a household with another family member, but not significant (95%CI = 0.89–1.66) p = 0.213.
USA					
High quality					
Chen et al. (2011) [41]	To determine the levels, sources and locations of and influential factors for exposure to Environmental Tobacco Smoke among pediatric patients.	397 participants– 82% African American	Cross sectional survey and children’s urine cotinine levels	ETS	Impact: adverse Smoking grandparents–around 30% major source of ETS 40% ETS exposure in relatives’ homes.
USA					
Low quality					
Mashita et al. (2011) [46]	To investigate current smoking behaviour among rural South African 11–18 year olds.	1654 11–18 year olds 854 boys 800 girls from Ellisras Longitudinal Study (rural South African children)	Cross-sectional survey with cluster randomised sampling	Tobacco product use and habits, attitudes and beliefs	Impact: adverse Children reported that grandparents influenced them to buy tobacco products: 33% of 11–12 year olds; 10% of 13–14 year olds; 15% of 15–16 year olds; 22% of 17–18 year olds.
South Africa					
High quality					
Robinson et al. (2011) [47]	How are positive messages about the need to protect children from tobacco smoke transmitted and discussed by adults, and how do they attempt to extend the protection of children outside their own household into that of others.	Phase 1–50 smokers and non-smokers living with smokers Phase 2–9 relatives of Phase 1 participants	Qualitative interviews	ETS	Impact: beneficial Some grandparents voluntarily change their smoking behaviour to protect children, whilst others do so when requested by parents.
UK (Scotland)					
High quality			Thematic analysis (applied aspects of social theory)	Applied aspects of social theory	
Carlsson et al. (2013) [45]	To provide nurses with new methods for motivating & supporting parents in their efforts to protect children from ETS	22 Child Healthcare Centre nurses recruited 86 families & children with at least one smoking parents. 72 families completed study.	Intervention–nurses using motivational interviewing skills & facilitating a dialogue with parents. Directing to websites	ETS	Impact: adverse Small mentions Higher urinary cotinine levels due to time children spend with grandparents.
Sweden					
Medium quality					
Thiangtham et al. (2013) [48]	Exploring and understanding the experiences of women smokers as well as the conditions and the family/social context of Thai society.	25 Thai women smokers in Bangkok & peripheral areas (14–66 years).	Focus groups and indepth interviews	Smoking	Impact: adverse Influence of grandparents in beginning smoking for children–rolling cigarettes.
Thailand					
Medium quality			Thematic analysis		
Escario & Wilkinson (2015) [49]	To analyse the extent to which parent and grandparent smoking simultaneously influences adolescent smoking.	32, 234 high school students aged 14–18.	Cross-sectional survey	Smoking status and consumption	Impact: beneficial/ no impact Having a cohabiting grandparent who smokes reduced the likelihood that adolescents smoke AOR 0.797 (95% CI = 0.645–0.985), but had no impact on smoking consumption levels.
Spain					
Medium quality				Social Learning Theory	
Mao (2015) [50]	To explore the role of mothers’ of young children in regulating family men’s smoking.	16 mothers, 5 grandmothers, 4 fathers, 4 grandfathers from 22 rural Chinese families with children under 6 years of age.	Ethnographic study (indepth interviews and observations)	Second hand smoke exposure	Impact: mixed Whilst grandfathers would smoke around children, grandmothers often intervened to prevent this.
China					
Medium quality				Theories of gender inequality	
Duarte et al. (2016) [51]	To investigate smoking influences in-home across three generations.	32, 234 high school students aged 14–18.	Cross-sectional survey	Smoking	Impact: adverse Increased likelihood of adolescents smoking if they live with a smoking grandparent AOR = 1.30 (95% CI = 1.07–1.57).
Spain					
				Social Learning Theory	
Medium quality					
Wang et al. (2017) [52]	To examine the association between household composition and children’s SHS exposure at home.	7911 3–11 year old children (parent report) from 2005, 2009 and 2013.	Repeated cross-sectional survey	Second hand smoke exposure	Impact: adverse Children living in households with grandparents greater likelihood of SHS exposure AOR 1.44 (95% CI = 1.23–1.69).
China (Taiwan)					
Medium quality					
Profe & Wild (2017) [37]	To investigate the independent and combined contributions of mother, father and closest grandparent involvement to the substance use of adolescents.	512 adolescents in grades 8 and 9 in two public high schools in Cape Town.	Cross sectional survey	Smoking Marijuana use	Impact: no impact Grandparent involvement not significantly related to smoking or marijuana use.
South Africa					
Medium quality					


### Weight, diet and physical activity

#### Geography

The majority of weight, diet and physical activity studies were from western countries, including the USA [54, 56, 61, 62, 71, 75, 77, 79, 81–83, 85, 86, 89], UK [55, 59, 63, 69, 87], Australia [72, 73, 80], Canada [70], Norway [64], Hungary [90], Poland [84], and two reviews reported on studies from a number of different countries [32, 60]. Seven studies reported results from China [52, 53, 57, 58, 67, 74, 88], and four studies from Japan [65, 66, 68, 76], reflecting three generational living in these two countries. A single study reported results from Egypt [78].

#### Study designs

The majority of studies contained qualitative information (n = 22) and three studies were reviews [32, 53, 60], one of which provided a review of Chinese language studies [53]. Of the qualitative studies, 13 included data collected from indepth interviews [58, 64, 71–75, 78, 80, 85, 86, 88, 90], 11 included data collected from focus group discussions [54, 56, 58, 69, 70, 72, 77, 79, 80, 82, 89] and one from a ethnographic study [84]. Quantitative studies included a mixture of cross-sectional [52, 57, 58, 61, 65, 81, 87, 88, 90], longitudinal [55, 59, 62, 63, 66, 68, 76, 83] and a case control design [67].

#### Participants

Study sample sizes ranged from 7 [75] to 300 [69] for qualitative studies, and from 62 [81] to 43046 [68] for quantitative studies. Although some studies focused on low income groups, the majority included participants of mixed socio-economic status, generally measured by income, educational level or occupation. Studies predominantly gathered data from parents [52, 54–59, 61–63, 65–68, 70–90] (n = 36), with information gathered from grandparents in 15 studies [58, 71, 72, 74, 77, 78, 81, 82, 84–90], children in seven [68, 69, 72, 77, 80, 84, 90], from health providers in one study [78], and school staff in two studies [58, 84].

#### Study aims

Nineteen studies had a specific aim of investigating the influence of grandparents on their grandchildren’s diet, physical activity or weight outcomes [32, 52, 58, 61, 63, 65, 66, 68, 71, 74, 75, 77, 81, 83, 85–88, 90]. These studies included a mix of qualitative and quantitative approaches. Three studies were literature reviews. One aimed to provide an overview of the evidence on interventions to reduce overweight and obesity in children within China [53]. The second aimed to synthesise qualitative literature on parental perceptions around weight-related behaviours [60]. The third aimed to gather and synthesise research findings on the effects of grandparent involvement on children’s physical health outcomes [32]. Other studies’ aims included investigating a range of factors (with grandparents included as one of a number) that might impact on children’s weight or weight-related behaviours, and were all quantitative [55, 57, 59, 62, 67, 76]. The remaining studies were qualitative and sought to gain a general understanding or additional insight into general weight, diet and food related issues in children [54, 56, 64, 69, 70, 72–74, 78–80, 82, 84, 89].

#### Theoretical framework

Five studies outlined or used a theoretical framework to guide and/or analyse their investigation. Goh et al. [88] used the Intergenerational Parenting Coalition approach to guide the study, that is the recognition that three-generational living forms part of China’s embedded cultural context, and that the interactions between generations are dialectical in influence. Sowan and Stember [62] drew on the Web of Causation Model [91] to examine how parental characteristics can impact children’s risk of obesity. This model focuses on risk and the interrelationships between risk factors. Styles et al. [82] used the socio-ecological approach [92] (the different levels that impact on an individuals’ health practices) to examine parents’ and grandparents’ concerns around obesity prevention, classifying them around intrapersonal, interpersonal, organizational and environmental levels. Boni [84] drew on Practice Theory in an ethnographic study that examined children’s food cultures in post-Soviet Poland, whilst Eli et al. [71] used a conceptual framework of familial homeostasis to discuss intergenerational feeding of children.

#### Study results

Weight—The evidence was strongest for grandparents having an adverse impact on children’s weight outcomes. No studies found a solely beneficial impact. Eight studies of the 17 studies found grandparents to have an adverse impact. Three studies found a mixed impact [32, 61, 64], and four found grandparents to have both adverse or no impacts for various relevant outcomes [58, 66–68]. Two studies found no impact [55, 62].

Diet—Similar to weight, grandparents overall appeared to have an adverse impact on their grandchildren’s diets [69, 70, 72, 73, 76, 78–80, 85, 88, 89], with an additional four studies reporting both adverse/no impacts [58, 65, 66, 83]. Nine studies reported mixed impacts [32, 71, 74, 75, 81, 82, 84, 86, 87]. One study found mixed/ no impact of grandparents [61]. Kaplan et al. [77] was the only study to find a solely beneficial impact of grandparents in managing and encouraging healthy eating in their grandchildren.

Physical activity—The evidence relating to children’s activity was less conclusive than for diet and weight, however, there was still greater evidence that grandparents’ actions had an adverse impact on grandchildren’s outcomes. Four studies identified adverse outcomes [52, 58, 76, 79], one study found mixed outcomes [71], one adverse/no impact [58], and a single study found no impact [65]. Lako [90] was the only study to find beneficial outcomes. They described both grandparents’ practical and support roles in transporting grandchildren to sporting activities, and actively cheering them on.

#### Study quality (see S2–S4 Tables)

For weight, diet and physical activity quantitative studies, seven were rated as high quality [55, 59, 62, 63, 65, 76, 83], demonstrating unbiased and externally valid results; seven were rated as medium quality [52, 57, 58, 66–68, 87], and three as low [61, 81, 90]. Low quality studies tended to have unreliable measures, low sample sizes and/or did not control adequately for confounding factors. Eight qualitative studies were rated as high quality [56, 58, 70, 71, 73, 75, 79, 86], ten were medium [54, 64, 72, 74, 78, 82, 84, 85, 88, 89] and four were low quality [69, 77, 80, 90]. Low quality studies had not adequately described the study context, the researchers’ roles, used reliable methods or conducted and/or reported the results of a rigorous analysis. These studies tended to inadequately report ethical procedures and approvals. All three review studies were rated as medium quality [32, 53, 60], with the search and inclusion of studies strong.

#### Thematic synthesis

Thematic analysis of extracted weight related qualitative data identified two broad themes describing grandparent impact: 1) Influence on family relationships and 2) Grandchildren’s diet. A more specific theme on physical activity was also identified. All studies reported some adverse impacts, with parents and grandparents reporting divergent views on appropriate eating behaviour [54, 56, 58, 64, 69, 70, 73–75, 78–80, 82, 84, 85, 88, 89]. This included the type of food provided, for example, high sugar or fat foods, or providing too much food. Parents reported feeling frustrated and undermined, and described these practices as ‘spoiling’ grandchildren. The need to rely on grandparents for childcare often resulted in grandparents’ practices prevailing [73, 75], however in other instances, grandparents reported that they followed parents’ rules. Johnson et al. [75] and Eli et al. [71] reported both adverse (eg, disconnected, ambivalent) and beneficial influences on family relationships. Where relationships were disconnected or ambivalent, mothers described rejecting some or all of the food practices that were modelled by grandmothers. These parents wished to provide their children with healthier foods, or to change mealtime practices, such as the rule that children must clear their plate. Conversely grandparents could also reject parents’ healthier food practices. Beneficial relationships were described through the intergenerational transmission of cooking skills and practices, but also through grandmothers being mindful of parents’ wish for children to eat healthily.

Four different roles around grandchildren’s diets were identified for grandparents’, (1) Buying and preparing food 2) Excessive and non-recommended feeding 3) Food as control/love 4) Promoting healthy food choices. 1) Buying and preparing food—A number of studies described grandparents as a source of support for parents either in buying, preparing and cooking food [71, 72, 74, 86, 88]. While the preparation of meals from scratch with fresh ingredients could be seen as a positive, this was undermined by the role grandparents could play in overfeeding children or feeding less healthy foods [71, 74, 79, 82, 85]. 2) Excessive and non-recommended feeding—Jiang et al. [74] and Li et al. [58] described grandparents’ excessively feeding children as a form of nurturing, where grandparents believed that overfeeding and excess weight were signs of health, and that this was a response to poverty and hunger experienced by grandparents in their youth. The feeding of non-recommended foods was demonstrated by grandmothers in Egypt, where they believed that children needed to eat ‘light’, sugary foods to thrive [78]. 3) Food as control/love—Related to overfeeding was grandparents using food to demonstrate their love for their grandchild [71, 74, 84]. This included practices such as physically feeding children who were capable of carrying this out for themselves, or providing foods prohibited by parents. Grandparents also said they used food a means through which to control grandchildren’s behaviour and to reward them for achievements. Roberts and Pettigrew [80] found that Australian parents reported that grandparents provided unhealthy food as a strategy to create a stronger bond between them and their grandchildren. Strategies to reduce tensions between parents and grandparents were not discussed in any studies. 4) Promoting healthy food choices—This theme was described in two studies [75, 77], and involved grandparents engaging with children in interactive ways to promote healthy eating, such as through humour, or by involving them in meal planning and preparation.

A final theme was grandparents’ impact on children’s activity-related practices. These included, physical activity, sedentary behaviour and screen time, and were discussed in three studies [58, 71, 90]. There was no overall direction in which grandparents appeared to impact these practices. Some grandparents put limits on children’s screen time, whist others allowed the same access as that provided at home. Physical activity levels appeared to be related to whether grandparents were active themselves, or whether there was appropriate space where children could be active. Grandparents were supportive of children’s participation in physical activity, and often enabled it through facilitating children’s access to spaces in which they could carry this out. In contrast to food, there appeared to be less tension in relation to parenting practices around activity.

### Tobacco studies

#### Geography

Similar to weight-related studies, the majority of tobacco studies drew on data from developed countries. Four studies were from the USA [38–41], three from China [42, 50, 52], two each from Spain [49, 51], South Africa [37, 46] and Sweden [44, 45], and one each from the UK [47], Czech Republic [43] and Thailand [48].

#### Study designs

Four studies reported qualitative findings [39, 47, 48, 50], Carlsson et al. [45] reported on the results of an intervention with parents around secondhand smoke (SHS), and the remaining studies were cross-sectional surveys [37, 38, 40–44, 46, 49, 51, 52].

#### Participants

Study sample sizes for the qualitative studies were 20 parents from low income families [39], 50 smokers and non smokers living with smokers and nine of their relatives [47], interviews with 22 Chinese families (n = 16 mothers, 5 grandmothers, 4 fathers, 4 grandfathers) [50], and 25 women smokers [48]. Quantitative studies ranged in study size from 174 [38] to 46,982 [40]. The majority of quantitative studies included representative or random samples. Parents were interviewed in seven studies [38–41, 47, 50, 52], and children in seven studies [37, 42, 43, 46, 48, 49, 51]. Two studies carried out research with grandparents [47, 50]. One study interviewed child healthcare nurses [44].

#### Study aims

Four studies examined the impact of grandparents on children’s smoking, or their exposure to secondhand smoke [37, 49–51]. Other studies aimed to examine the relationship between children’s levels of SHS exposure [38–41, 44, 47, 52] or respiratory ill health [39, 42] and a range of possible environmental exposures (including grandparents), or potential influences on children’s smoking behaviour [43, 46, 48]. The intervention study evaluated new methods for nurses to support parents in protecting their children from SHS [45].

#### Theoretical framework

Four studies made reference to theory. Robinson et al. [47] analysed in-depth interview data drawing from aspects of social theory, though this was not specified further. Escario and Wilkinson and Duarte et al. (using data from the same survey) used social learning theory to frame their cross-sectional analyses of in-home influences on children’s smoking practices. Mao [50] used theories of gender inequality to investigate the role of mothers’ and grandmothers’ in regulating grandfathers’ smoking around young children.

#### Study results

Nine studies out of 16 found that there was an adverse impact of grandparents on children’s smoking or exposure to SHS [38, 39, 41, 44–46, 48, 51, 52]. Three additional studies found a mix of adverse impacts for some outcomes and no impact for others [40, 42, 43]. Living with a grandparent who smoked was problematic [37, 40, 42, 49–51], as was visiting grandparents who smoked [38, 39, 41, 44, 45]. Mao [50] found that living with grandfathers who smoked increased children’s secondhand smoke exposure, but that living with grandmothers was protective. Profe and Wild [37] found no impact of grandparent involvement on smoking. Robinson et al. [47] identified a beneficial impact of grandparents, with participants reporting that becoming a grandparent had prompted grandparent smokers to reassess their habits, and to no longer smoke indoors when their grandchildren were present. Escario and Wilkinson [49] found that living with a grandparent reduced the likelihood that a child would smoke, but had no impact on the consumption levels of children who did smoke.

#### Study quality (see S5–S7 Tables)

For quantitative tobacco studies, three were rated as high quality [40, 42, 46], four were rated as medium quality [44, 49, 51, 52] and four as low [37, 38, 41, 43]. Low quality studies had low sample sizes or unrepresentative samples and/or did not control adequately for confounding. Two qualitative studies were rated as high quality [39, 47], and two medium quality [48, 50]. The single intervention study (a before/after design) was rated as medium quality [45], with weaknesses explicitly around analysis.

#### Thematic synthesis

The four qualitative studies provided only limited data for the thematic synthesis [39, 47, 48, 50]. The three themes were, 1) parents limiting grandparent access to grandchildren who smoke, 2) grandparents protecting children from SHS, and 3) grandparents acting as negative role models. For the first theme, parents described their inability to enforce rules around grandparents smoking in the home, and that this resulted in parents limiting grandparent interaction with grandchildren [39, 50]. In contrast Robinson et al. [47], found that the birth of grandchildren was a catalyst that encouraged grandparents to stop smoking completely, or to stop smoking indoors when their grandchildren were present. This was also true of some grandparents in Mao’s study of Chinese grandparents [50]. The third theme of grandparents acting as negative role models was discussed by Thai women [48] who described growing up around grandparents who smoked. As well as seeing smoking practices frequently, they became more involved through buying or rolling tobacco for grandparents. Women said they believed this early exposure and involvement in smoking practices partly explained their own smoking as an adult.

### Alcohol

A single study examined the impact of grandparents on children’s alcohol consumption (Table 4). Prof and Wilde [37] used cross-sectional data gathered from adolescents in South Africa to investigate whether grandparent involvement predicted use of alcohol. The results found no significant impact, and the study was rated as low quality.

## Discussion

This review has been the first to identify and synthesise evidence for the influence of grandparents on their grandchildren’s long term cancer risk factors. Results indicated that there was a sufficient evidence base for weight, diet, physical activity and tobacco studies to draw conclusions about grandparents’ impact. There was minimal evidence for alcohol and no evidence for sun exposure.

Evidence for weight, diet, physical activity and tobacco studies strongly suggest that grandparents had an adverse impact on their grandchildren’s health in these areas [52–54, 56, 57, 59, 60, 63, 67, 69, 70, 72–74, 76, 78–80, 85, 88, 89]. In the tobacco studies reviewed, grandparents smoked around grandchildren, did not comply with parents’ wishes regarding SHS, and role modelled negative behaviour which led to grandchildren taking up smoking [38–46, 48, 51, 52]. For weight-related studies, grandparents were characterised by parents as indulgent, misinformed and as using food as an emotional tool within their relationships with grandchildren [74, 79, 80, 82]. However, much of the evidence for these studies came from parents, with a relatively small number of studies representing grandparents’ perspectives [58, 71, 72, 74, 77, 78, 81, 82, 84–90]. Nevertheless, quantitative studies also provided evidence for an adverse impact, in some cases using objective measures [52, 55, 57, 59, 61–63, 66–68]. For example, Pearce et al.[59] found that children looked after in informal childcare, the majority of which was provided by grandparents, were more likely to be overweight. It is noteworthy that this relationship was only found in families where parents were described as socio-economically advantaged.

Studies that showed a beneficial impact highlighted that grandparents did not always undermine parents, and could play a role in promoting healthy eating practices [32, 57, 61, 64, 65, 71, 75, 77, 81, 82, 84, 86, 87, 90]. Robinson et al. [47] provided a high quality in-depth study of smokers, ex-smokers and their families, identifying grandparenthood as a pivotal point for behaviour change, either by deciding to stop smoking completely or stop smoking in the home. It was not possible to identify under what circumstances these beneficial impacts took place due to the heterogeneity of the included studies.

The results indicate a lack of theoretical rigour in most of the studies in this area. Only nine studies used or made reference to an explicit theoretical framework. These included the intergenerational parenting coalition [88], web of causation [62], socio-ecological models [82], social learning theory [49, 51], practice theory [84], familial homeostasis [71] and theories of gender inequality [50]. In identifying grandparents as impacting adversely on their grandchildren’s cancer risk factors, studies failed to take into account the wider context in which the results are to be understood. Grandparents are likely to be one of many influences on health outcomes, and are located at the interpersonal level of the socio-ecological impacts on health [92]. Indeed grandparenting exists within a complex social system in which it interacts with influences at a variety of levels in children’s lives. Few of the studies above discuss these in depth, but they suggest some potentially important influences, such as parents’ working patterns, societal norms and lack of other childcare options.

The studies included in the review do not take into account the more general beneficial role grandparents may play in their grandchildren’s lives. Grandparents may be better able to spend time with their grandchildren in ways that parents are unable to. This can help facilitate good social and emotional wellbeing in grandchildren, and therefore, any recommendation to limit grandparent interaction with their grandchildren would be misplaced. Instead, as suggested by results from the Growing Up in Scotland cohort studies [33], parenting advice and support needs to be broadened to encompass grandparents as well as parents. Grandparents’ roles must be recognised and practical steps put in place to facilitate optimal intergenerational parenting. In some studies, there were hints that tensions could arise between parents and grandparents, with little suggestion of how communication between generations could be enhanced to ensure that shared understandings around parenting could be realised. In addition, there were no interventions identified that sought to encompass grandparents as a potential mechanism through which to improve grandchildren’s diets. With the caring role of grandparents now being recognised within the UK legislation and benefits system, and the expectation that grandparents’ involvement in their grandchildren’s lives will only increase, there is a need for theoretically grounded interventions to be designed that include significant communication-based components.

### Strengths and limitations

This study has integrated the evidence-base on the impact of grandparents on grandchildren’s cancer risk factors. Cancer research has focused more on treatment of disease rather than the full range of factors that might play a role in cancer prevention over the life course. The review therefore took a broad approach to the types of evidence considered for syntheses, with qualitative literature synthesised through a thematic analysis of participant quotations and author analyses. A thorough quality appraisal also took place using appropriate tools for each of the study types included. A larger proportion of qualitative studies were rated as lower quality compared with quantitative studies.

While the review used a range of key databases to identify relevant articles, it did not ask authors or experts to identify additional studies, and did not include findings from the grey literature. In addition, non-English language studies were not included, which limits the applicability of the review findings across cultures. An additional limitation was that many studies contained only a limited description of grandparents’ impact, and/or provided little indication of the extent to which the amount of time grandchildren spent with grandparents was associated with more adverse outcomes or behaviours.

## Conclusion

The weight of the evidence within this review found that grandparents had an adverse impact on their grandchildren’s cancer risk factors. Future work should focus on realising the potential for grandparents to be a positive influence on their grandchildren’s health through the design of realistic, theoretically underpinned interventions. Interventions should ideally include components that aid facilitating family communication around areas of tension. The formative stages of this work should include the perspectives of both grandparents and grandchildren to enhance the likelihood of success.

## Supporting information

S1 Table Medline search terms. (DOCX)

S2 Table Observational and cohort study quality–weight, diet and physical activity studies. ++ Indicates that for that particular aspect of study design, the study has been designed or conducted in such a way as to minimise the risk of bias. + Indicates that either the answer to the checklist question is not clear from the way the study is reported, or that the study may not have addressed all potential sources of bias for that particular aspect of study design.—Should be reserved for those aspects of the study design in which significant sources of bias may persist. NR–Not reported—Should be reserved for those aspects in which the study under review fails to report how they have (or might have) been considered. NA–Not applicable—Should be reserved for those study design aspects that are not applicable given the study design under review.
(DOCX)

S3 Table Qualitative study quality–weight, diet and physical activity studies. IR–Inadequately reported. NR–Not reported.
(DOCX)

S4 Table Review study quality–weight, diet and physical activity studies. (DOCX)

S5 Table Observational and cohort study quality–tobacco studies. ++ Indicates that for that particular aspect of study design, the study has been designed or conducted in such a way as to minimise the risk of bias. + Indicates that either the answer to the checklist question is not clear from the way the study is reported, or that the study may not have addressed all potential sources of bias for that particular aspect of study design.—Should be reserved for those aspects of the study design in which significant sources of bias may persist. NR–Not reported—Should be reserved for those aspects in which the study under review fails to report how they have (or might have) been considered. NA–Not applicable—Should be reserved for those study design aspects that are not applicable given the study design under review.
(DOCX)

S6 Table Qualitative study quality–tobacco studies. NR–Not reported.
(DOCX)

S7 Table Intervention study quality–tobacco studies. (DOCX)
