# Supplementary material for: A systematic review of grandparents’ influence on grandchildren’s cancer risk factors
Source: PLoS One. 2017 Nov 14;12(11):e0185420. doi: 10.1371/journal.pone.0185420 (PMC5685489; doi:10.1371/journal.pone.0185420)
Supplement: S1 Table — (DOCX) [file pone.0185420.s001.docx]

| 1 | Grandparent*  "grand parent*"  Grandmother*  "grand mother*"  Grandfather*  "grand father*"  Grandchild*  "grand child*"  Grandaughter*  "grand daughter*"  Grandson*  "grand son*"  Grandmaternal  Intergenerational | |
| --- | --- | --- |
| 2 | **MESH**  Smoking  "Tobacco Use Disorder"  "Tobacco Smoke Pollution"  Diet  Meals  Food  Overweight  "Body Mass Index"  Pediatric  "Physical Activity"  Fruit  Vegetables  Alcohol Drinking  Binge Drinking  Alcoholic Intoxication  Sunbathing  Sunburn  Suntan  Sunscreening Agents | **Free text**  Diet*  "Second hand smoke"  Smoking  Smoke*  Fruit*  Vegetable*  Sugar  Tobacco  Feeding Dairy  Egg*  Meat  Cake*  Candy  Meal*  Overweight  Obesity  Obese  Snack*  Feed*  Food  Dinner  Breakfast  Lunch  Fed  Eat*  Nutrition*  Portion*  "body mass index"  BMI  Weigh*  "physical activity"  Soda  ETS (environmental tobacco smoke)  Sun*  Alcohol*  Confectionery  Fibre  SHS |
