# Supplementary material for: A systematic review of grandparents’ influence on grandchildren’s cancer risk factors
Source: PLoS One. 2017 Nov 14;12(11):e0185420. doi: 10.1371/journal.pone.0185420 (PMC5685489; doi:10.1371/journal.pone.0185420)
Supplement: S2 Table — ++ Indicates that for that particular aspect of study design, the study has been designed or conducted in such a way as to minimise the risk of bias. + Indicates that either the answer to the checklist question is not clear from the way the study is reported, or that the study may not have addressed all potential sources of bias for that particular aspect of study design.—Should be reserved for those aspects of the study design in which significant sources of bias may persist. NR–Not reported—Should be reserved for those aspects in which the study under review fails to report how they have (or might have) been considered. NA–Not applicable—Should be reserved for those study design aspects that are not applicable given the study design under review. (DOCX) [file pone.0185420.s002.docx]

| **Study and overall quality** | **Population** | **Method of selection of exposure (or comparison) group** | **Outcomes** | **Analyses** | **Summary** |
| --- | --- | --- | --- | --- | --- |
| Kagamimori et al. (1999) [76]  High | Well described: ++  Represents source population: ++  Represents eligible population: ++ | Selection bias: NR  Theory: ++  Confounding factors controlled: + | Reliable outcome measures & procedures: ++  Complete outcome measures: ++  Important outcomes assessed: ++  Follow-up time: NR | Sufficient power: NR  Multiple explanatory variables: +  Appropriate analytical methods: +  Precise associations: ++ | Unbiased results: +  Externally valid results: ++ |
| Sowan & Stember (2000) [62]  High | Well described: ++  Represents source population: ++  Represents eligible population: ++ | Selection bias: +  Theory: ++  Confounding factors controlled: + | Reliable outcome measures & procedures: ++  Complete outcome measures: ++  Important outcomes assessed: ++  Follow-up time: + | Sufficient power: NR  Multiple explanatory variables: ++  Appropriate analytical methods: ++  Precise associations: ++ | Unbiased results: +  Externally valid results: ++ |
| Hawkins et al. (2008) [55]  High | Well described: ++  Represents source population: ++  Represents eligible population: ++ | Selection bias: ++  Theory: ++  Confounding factors controlled: ++ | Reliable outcome measures & procedures: ++  Complete outcome measures: ++  Important outcomes assessed: ++  Follow-up time: ++ | Sufficient power: ++  Multiple explanatory variables: ++  Appropriate analytical methods: ++  Precise associations: ++ | Unbiased results: ++  Externally valid results: ++ |
| Speirs et al. (2009) [81]  Low | Well described: +  Represents source population: -  Represents eligible population: - | Selection bias: NR  Theory: -  Confounding factors controlled: - | Reliable outcome measures & procedures: +  Complete outcome measures: -  Important outcomes assessed: +  Follow-up time: NA | Sufficient power: -  Multiple explanatory variables: -  Appropriate analytical methods: +  Precise associations: - | Unbiased results: -  Externally valid results: - |
| Pearce et al. (2010) [59]  High | Well described: ++  Represents source population: ++  Represents eligible population: ++ | Selection bias: ++  Theory: ++  Confounding factors controlled: ++ | Reliable outcome measures & procedures: ++  Complete outcome measures: ++  Important outcomes assessed: ++  Follow-up time: ++ | Sufficient power: NR  Multiple explanatory variables: ++  Appropriate analytical methods: ++  Precise associations: ++ | Unbiased results: ++  Externally valid results: ++ |
| Watanabe et al. (2011) [65]    High | Well described: +  Represents source population: ++  Represents eligible population: + | Selection bias: NR  Theory: ++  Confounding factors controlled: - | Reliable outcome measures & procedures: +  Complete outcome measures: ++  Important outcomes assessed: ++  Follow-up time: NA | Sufficient power: NA  Multiple explanatory variables: ++  Appropriate analytical methods: ++  Precise associations: ++ | Unbiased results: +  Externally valid results: ++ |
| Li, Adab & Cheng (2013) [57]  Medium | Well described: ++  Represents source population: +  Represents eligible population: - | Selection bias: NR  Theory: +  Confounding factors controlled: + | Reliable outcome measures & procedures: +  Complete outcome measures: ++  Important outcomes assessed: ++  Follow-up time: NA | Sufficient power: NR  Multiple explanatory variables: ++  Appropriate analytical methods: ++  Precise associations: ++ | Unbiased results: +  Externally valid results: + |
| Pulgarón et al. (2013) [61]  Low | Well described: +  Represents source population: -  Represents eligible population: - | Selection bias: NR  Theory: NR  Confounding factors controlled: NR | Reliable outcome measures & procedures: -  Complete outcome measures: -  Important outcomes assessed: -  Follow-up time: NR | Sufficient power: -  Multiple explanatory variables: -  Appropriate analytical methods: +  Precise associations: + | Unbiased results: -  Externally valid results: - |
| Tanskanen (2013) [63]  High | Well described: ++  Represents source population: ++  Represents eligible population: ++ | Selection bias: ++  Theory: ++  Confounding factors controlled: ++ | Reliable outcome measures & procedures: ++  Complete outcome measures: ++  Important outcomes assessed: ++  Follow-up time: ++ | Sufficient power: ++  Multiple explanatory variables: ++  Appropriate analytical methods: ++  Precise associations: ++ | Unbiased results: ++  Externally valid results: ++ |
| Wasser et al. (2013) [83]  High | Well described: ++  Represents source population: ++  Represents eligible population: ++ | Selection bias: NR  Theory: ++  Confounding factors controlled: ++ | Reliable outcome measures & procedures: +  Complete outcome measures: ++  Important outcomes assessed: ++  Follow-up time: + | Sufficient power: ++  Multiple explanatory variables: ++  Appropriate analytical methods: ++  Precise associations: + | Unbiased results: ++  Externally valid results: ++ |
| Farrow (2014) [87]  Medium | Well described: ++  Represents source population: ++  Represents eligible population: ++ | Selection bias: NR  Theory: ++  Confounding factors controlled: - | Reliable outcome measures & procedures: +  Complete outcome measures: ++  Important outcomes assessed: ++  Follow-up time: NA | Sufficient power: -  Multiple explanatory variables: +  Appropriate analytical methods: +  Precise associations: ++ | Unbiased results: +  Externally valid results: + |
| Lako (2014) [90]  Low | Well described: -  Represents source population: -  Represents eligible population: - | Selection bias: NR  Theory: -  Confounding factors controlled: - | Reliable outcome measures & procedures: -  Complete outcome measures: -  Important outcomes assessed: -  Follow-up time: NA | Sufficient power: NR  Multiple explanatory variables: -  Appropriate analytical methods: -  Precise associations: - | Unbiased results: -  Externally valid results: - |
| Li et al. (2015) [58]  Medium | Well described: -  Represents source population: -  Represents eligible population: - | Selection bias: -  Theory: -  Confounding factors controlled: + | Reliable outcome measures & procedures: ++  Complete outcome measures: ++  Important outcomes assessed: ++  Follow-up time: NA | Sufficient power: -  Multiple explanatory variables: ++  Appropriate analytical methods: ++  Precise associations: ++ | Unbiased results: +  Externally valid results: - |
| Sata et al. (2015) [66]  Medium | Well described: ++  Represents source population: ++  Represents eligible population: ++ | Selection bias: +  Theory: +  Confounding factors controlled: ++ | Reliable outcome measures & procedures: -  Complete outcome measures: ++  Important outcomes assessed: ++  Follow-up time: ++ | Sufficient power: +  Multiple explanatory variables: +  Appropriate analytical methods: +  Precise associations: ++ | Unbiased results: +  Externally valid results: + |
| Zong et al. (2015) [67]  Medium | Well described: -  Represents source population: +  Represents eligible population: + | Selection bias: +  Theory: -  Confounding factors controlled: + | Reliable outcome measures & procedures: ++  Complete outcome measures: ++  Important outcomes assessed: ++  Follow-up time: NA | Sufficient power: +  Multiple explanatory variables: +  Appropriate analytical methods: +  Precise associations: ++ | Unbiased results: +  Externally valid results: + |
| Wang & Qi (2016) [52]  Medium | Well described: +  Represents source population: -  Represents eligible population: NR | Selection bias: NR  Theory: +  Confounding factors controlled: + | Reliable outcome measures & procedures: ++  Complete outcome measures: ++  Important outcomes assessed: +  Follow-up time: NA | Sufficient power: -  Multiple explanatory variables: +  Appropriate analytical methods: +  Precise associations: + | Unbiased results: +  Externally valid results: - |
| Ikeda et al. (2017) [68]  Medium | Well described: +  Represents source population: ++  Represents eligible population: ++ | Selection bias: +  Theory: +  Confounding factors controlled: + | Reliable outcome measures & procedures: -  Complete outcome measures: ++  Important outcomes assessed: ++  Follow-up time: NA | Sufficient power: +  Multiple explanatory variables: ++  Appropriate analytical methods: ++  Precise associations: ++ | Unbiased results: +  Externally valid results: + |
