# Supplementary material for: A systematic review of grandparents’ influence on grandchildren’s cancer risk factors
Source: PLoS One. 2017 Nov 14;12(11):e0185420. doi: 10.1371/journal.pone.0185420 (PMC5685489; doi:10.1371/journal.pone.0185420)
Supplement: S3 Table — IR–Inadequately reported. NR–Not reported. (DOCX) [file pone.0185420.s003.docx]

| **Study & overall assessment** | **Appropriate approach** | **Clear aim** | **Design defens-ible** | **Data collection** | **Researcher role clear** | **Context described** | **Methods reliable** | **Rigorous analysis** | **Rich data** | **Reliable analysis** | **Findings convincing** | **Findings relevant** | **Conclusions adequate** | **Ethics clear** |
| --- | --- | --- | --- | --- | --- | --- | --- | --- | --- | --- | --- | --- | --- | --- |
| Auld & Morris (1994) [85]  Medium | Not sure | Yes | Not sure | Yes | No | Yes | Not sure | NR | No | Not sure | Yes | Yes | No | No |
| Dixey et al. (2001) [69]  Low | Not sure | Yes | Not sure | IR | No | Yes | Not sure | No | NR | NR | Not sure | Yes | Not sure | NR |
| Jain et al. (2001) [56]  High | Yes | Yes | Yes | Yes | No | Yes | Yes | Yes | Yes | Yes | Yes | Yes | Yes | Yes |
| Green et al. (2003) [72]  Medium | Yes | Yes | Yes | Yes | No | No | Not sure | NR | NR | NR | Not sure | Yes | Yes | NR |
| Jiang et al. (2007) [74]  Medium | Yes | Yes | Yes | IR | No | No | No | NR | No | NR | Not sure | Yes | Yes | Yes |
| Kaplan et al. (2006) [77]  Low | Yes | Yes | Yes | Yes | Unclear | Yes | No | Not sure | Yes | Yes | No | Yes | Yes | No |
| Styles et al. (2007) [82]  Medium | Yes | Mix | Yes | Yes | No | Yes | Yes | NR | Yes | Yes | Yes | Yes | Yes | Yes |
| Dwyer et al. (2008) [70]  High | Yes | Yes | Yes | Yes | No | Yes | Yes | Yes | Yes | Yes | Yes | Yes | Yes | Yes |
| Lindsay et al (2009) [79]  High | Yes | Yes | Yes | Yes | No | Yes | Yes | Yes | Yes | Yes | Yes | Yes | Yes | Not sure |
| Johnson et al. (2010) [75]  High | Yes | Yes | Yes | Yes | No | Yes | Yes | Yes | Yes | Yes | Yes | Yes | Yes | Yes |
| Roberts & Pettigrew (2010) [80]  Low | Yes | Yes | Not sure | IR | No | No | Not sure | Not sure | No | NR | Not sure | Yes | Not sure | NR |
| Glassman et al. (2011) [54]  Medium | Yes | Yes | Yes | IR | No | Not sure | Not sure | Yes | NR | Yes | Yes | Yes | Yes | Yes |
| Goh et al. (2013) [88]  Medium | Yes | Yes | Yes | Yes | No | Not sure | Yes | Yes | Yes | Yes | Yes | Yes | Yes | Yes |
| Toftemo et al. (2013) [64]  Medium | Yes | Yes | Yes | Yes | No | No | Yes | NR | NR | Yes | Yes | Yes | Yes | NR |
| Hoare et al. (2014) [73]  High | Yes | Yes | Yes | Yes | No | Yes | Yes | Yes | Yes | Yes | Yes | Yes | Yes | Yes |
| Lako (2014) [90]  Low | Not sure | No | No | IR | No | No | No | No | No | NR | No | Partially | No | No |
| Boni (2015) [84]  Medium | Yes | Yes | Yes | IR | No | Yes | Yes | NR | Yes | NR | Yes | Yes | Yes | NR |
| Kavle et al. (2015) [78]  Medium | Yes | Yes | Yes | Yes | No | Yes | Yes | Yes | NR | Yes | Yes | Yes | Yes | NR |
| Li et al. (2015) [58]  High | Yes | Yes | Yes | Yes | No | Yes | Yes | Yes | No | Yes | Yes | Yes | Yes | NR |
| Mena & Gorman (2015) [89]  Medium | Yes | Yes | Yes | Yes | No | Yes | Yes | Yes | NR | Not sure | Yes | Yes | Yes | Yes |
| Eli et al. (2015) [71]  High | Yes | Yes | Yes | Yes | No | Yes | Yes | Yes | Yes | Yes | Yes | Yes | Yes | Yes |
| Eli et al. (2017) [86]  High | Yes | Yes | Yes | Yes | No | Yes | Yes | Yes | Yes | Yes | Yes | Yes | Yes | Yes |
