# Supplementary material for: A systematic review of grandparents’ influence on grandchildren’s cancer risk factors
Source: PLoS One. 2017 Nov 14;12(11):e0185420. doi: 10.1371/journal.pone.0185420 (PMC5685489; doi:10.1371/journal.pone.0185420)
Supplement: S4 Table — (DOCX) [file pone.0185420.s004.docx]

| **Study & overall assessment** | **Focused question** | **Relevant studies included** | **Search rigorous** | **Study quality assessed & reported** | **Method described & appropriate** |
| --- | --- | --- | --- | --- | --- |
| Gao et al. (2007) [53]  Medium | No | Yes | Yes | No | Yes |
| Pocock et al. (2010) [60]  Medium | Yes | Yes | No | Yes | Yes |
| Pulgaron et al. (2016) [32] | Yes | Yes | No | No | Yes |
| Medium |  |  |  |  |  |
