# Supplementary material for: A systematic review of grandparents’ influence on grandchildren’s cancer risk factors
Source: PLoS One. 2017 Nov 14;12(11):e0185420. doi: 10.1371/journal.pone.0185420 (PMC5685489; doi:10.1371/journal.pone.0185420)
Supplement: S6 Table — NR–Not reported. (DOCX) [file pone.0185420.s006.docx]

| **Study & overall assessment** | **Appropriate approach** | **Clear aim** | **Design defens-ible** | **Data collection** | **Researcher role clear** | **Context described** | **Methods reliable** | **Rigorous analysis** | **Rich data** | **Reliable analysis** | **Findings convincing** | **Findings relevant** | **Conclusions adequate** | **Ethics clear** |
| --- | --- | --- | --- | --- | --- | --- | --- | --- | --- | --- | --- | --- | --- | --- |
| Robinson et al. (2010) [47]  High | Yes | Yes | Yes | Yes | No | Yes | Yes | Yes | Yes | Yes | Yes | Yes | Yes | NR |
| Youssey (2007) [39]  High | Yes | Yes | Yes | Yes | Yes | Not sure | Yes | Yes | Yes | Yes | Yes | Yes | Yes | Yes |
| Thiangtham et al. (2013) [48]  Medium | Yes | Mixed | No | Yes | Yes | Yes | Yes | NR | NR | NR | Yes | Yes | No | Yes |
| Mao (2014) [50]  Medium | Yes | Yes | Yes | Yes | No | Yes | Yes | Yes | Yes | No | Yes | Yes | Yes | No |
