# Supplementary material for: A systematic review of grandparents’ influence on grandchildren’s cancer risk factors
Source: PLoS One. 2017 Nov 14;12(11):e0185420. doi: 10.1371/journal.pone.0185420 (PMC5685489; doi:10.1371/journal.pone.0185420)
Supplement: S7 Table — (DOCX) [file pone.0185420.s007.docx]

| **Study** | **Population** | **Method of selection of exposure (or comparison) group** | **Outcomes** | **Analyses** | **Summary** |
| --- | --- | --- | --- | --- | --- |
| Carlsson et al. (2013) [45]  Medium | Well described: ++  Represents source population: ++  Represents eligible population: + | Selection bias: NA  Intervention well described: ++  Allocation concealed: NA  Blinding: NA  Exposure adequate: NR  Contamination low: NA  Similar other interventions: NA  All participants accounted for: ++ | Reliable outcome measures: ++  Complete outcome measures: ++  Important outcomes assessed: ++  Outcomes relevant: ++  Follow-up times similar: NA  Follow-up time meaningful: ++ | Similarities at baseline: NA  Intention to treat analysis: NA  Sufficient power: NR  Estimates of effect size: NR  Appropriate analytical methods: +  Precision: - | Unbiased results: +  Externally valid results: - |
